# Adherence to the Mediterranean Diet and Determinants Among Pregnant Women: The NELA Cohort

**DOI:** 10.3390/nu13041248

**Published:** 2021-04-10

**Authors:** Clara Suárez-Martínez, Genoveva Yagüe-Guirao, Marina Santaella-Pascual, Patricia Peso-Echarri, Jesús Vioque, Eva Morales, Luis García-Marcos, Carmen Martínez-Graciá, The NELA Study Group

**Affiliations:** 1 Biomedical Research Institute of Murcia (IMIB-Arrixaca), 30120 Murcia, Spain; clara.suarez@um.es (C.S.-M.); gyague@um.es (G.Y.-G.); marinasp@um.es (M.S.-P.); patri.peso@um.es (P.P.-E.); evamorales@um.es (E.M.); lgmarcos@um.es (L.G.-M.); 2 Food Science and Nutrition Department, Veterinary Faculty, CEIR Campus Mare Nostrum (CMN), University of Murcia, 30100 Murcia, Spain; 3 Microbiology Service, Virgen de La Arrixaca University Clinical Hospital, CEIR Campus Mare Nostrum (CMN), University of Murcia, 30120 Murcia, Spain; 4 Health and Biomedical Research Institute of Alicante (ISABIAL-UMH), 46020 Alicante, Spain; vioque@umh.es; 5 Spanish Consortium for Research on Epidemiology and Public Health (CIBERESP), 28029 Madrid, Spain; 6 Department of Public Health Sciences, University of Murcia, 30100 Murcia, Spain; 7 Paediatric Allergy and Pulmonology Units, Virgen de La Arrixaca University Clinical Hospital, ARADyAL Allergy Network, CEIR Campus Mare Nostrum (CMN), University of Murcia, 30120 Murcia, Spain

**Keywords:** dietary pattern, Mediterranean diet, healthy diet, pregnancy, lifestyle, sociodemographic factors

## Abstract

The Mediterranean diet represents one of the most studied dietary patterns; however, there is no single tool for measuring the grade of adherence and no single set of criteria for adapting these indices to pregnant women. We characterized the adherence to the Mediterranean diet (MDA) of pregnant women participating in the NELA (Nutrition in Early Life and Asthma) cohort and identified the sociodemographic determinants and lifestyle habits associated with a higher risk of a low MDA. Maternal diet during gestation was assessed by a validated Food Frequency Questionnaire (FFQ) (*n* = 665). We estimated the Relative Mediterranean Diet score (rMED), Alternative Mediterranean Diet score (aMED), and the Alternate Healthy Eating Index-2010 (AHEI-2010). Multivariate regression models were performed to identify the sociodemographic and lifestyle factors associated with each index. Mothers with a lower age and more previous deliveries had a greater probability of low MDA (*p* < 0.05). For the aMED index only, mothers with university education and/or who practiced sport activities for two or more hours per week had a lower probability of a low MDA (*p* < 0.01). The three indices classified the NELA cohort as having a medium level of adherence. These results may be improved by designing intervention strategies and dietary recommendations for both maternal and offspring health.

## 1. Introduction

The period of prenatal life is considered to be a critical window for both the mother’s and offspring’s health; in particular, the maternal diet during pregnancy has been proposed as one of the prenatal factors that has long-term implications and influences on the development of the placenta [1] and on the risk of developing gestational diabetes [2]. The prenatal diet is also associated with complications at birth including premature birth [3,4], pre-eclampsia [5], low birth weight [3], and it may influence the correct development and response of the foetal immune system and consequently the risk of developing allergies or asthma in childhood [6,7,8,9,10]. In addition, during pregnancy, maternal Mediterranean diet adherence (MDA) may promote behavioral and emotional wellbeing in children [11]. Traditionally, the isolated effects of foods or specific nutrients consumed during pregnancy on health and their possible role in the development of diseases in offspring have been investigated. However, this type of analysis can omit relevant information and be inaccurate since foods are consumed together in the context of a diet, creating synergies between them [12,13].

The Mediterranean diet (MD) and Mediterranean diet adherence (MDA) have been investigated due to its beneficial effects and protective role against diseases, demonstrated in numerous high-quality studies, reviews [6,14,15], and meta-analyses [16,17,18,19], making it the most widely studied and evidence-based dietary approach to healthy eating and disease prevention [17,19,20]. One of the last and most correct definitions of the MD during pregnancy is the one described by Amati et al. [21] in their review in 2019. The authors described the MD as a “diet characterized by a high intake of fruits, vegetables, whole grain cereals and bread, legumes, fish and nuts; low-to-moderate consumption of dairy products and eggs, and limited amounts of red meat and red wine. It is low in saturated fats and high in antioxidants, fibre and mono and polyunsaturated fatty acids (MUFAs and PUFAs) mainly derived from extra virgin olive oil and oily fish (n-3 PUFAs)”. Beyond nutritional guidelines, the MD represents a balanced and healthy lifestyle that includes physical activity, adequate rest, traditional and simple ways of cooking, and sociability at the table as the main habits registered in the latest available edition of the Pyramid of MD, published by the Mediterranean Diet Foundation [22]. Olmeda-Requena et al. in 2014 [23] carried out a research to study the factors associated with a low adherence to an MD pattern in healthy women and concluded that a younger age, a low social class, a low educational level, and an unhealthy lifestyle (smoking and lack of exercise) were associated with a low MDA.

Trichopoulou et al. in 2003 [24] were the first researchers to quantify MDA using a 10-point numerical scale, which is the well-known Mediterranean Diet Score (MDS). They reported an inverse association between the score obtained and total mortality. The higher the MDA, the lower the mortality rate, both from coronary heart disease and cancer. In relation to the numerous indices that have been developed to measure the MDA in the adult population, Olmedo-Requena et al. [25] studied the degree of correlation between the five different indices that have been developed to date and applied to the same population (healthy adults), concluding that concordance between them (including the Relative Mediterranean Diet score (rMED) and Alternative Mediterranean Diet score (aMED)) was moderate or low. The authors reported the existence of a different classification of the subjects due to variations in the food groups included. 

Furthermore, in most of the cohorts that study the pattern of adherence to the MD of adults or pregnant women, a single index has been used, and there is no consensus when applying one or the other index. To date, no comparison has been made of the two most widely used scores (rMED and aMED), applied to the same population of pregnant women or the sociodemographic and lifestyle factors that influence this degree of adherence to check if the same conclusions are reached—this being a complementary objective of this work.

The aims of this study were to evaluate the adherence to the MD in pregnant women of the NELA study (Nutrition in Early Life and Asthma), a prospective birth cohort study established in the Mediterranean Region of Murcia (Spain), and to identify lifestyles and sociodemographic factors associated with a low MDA.

## 2. Materials and Methods

### 2.1. Study Population

The Nutrition in Early Life and Asthma (NELA) birth cohort study recruited 738 pregnant women between 2015 and 2018 in Murcia, a south-eastern Mediterranean region of Spain (www.nela.imib.es). The main objective of NELA is to unravel the developmental origins and mechanisms of asthma and allergy. Recruitment took place at the time of the control ultrasound at 20 weeks of gestation at the Maternal–Fetal Unit of the Virgen de la Arrixaca University Hospital. The inclusion criteria were women from Health Area I and certain districts of Health Areas VI and VII of the Region of Murcia; planning to live in the area of study for at least 2 years; the intention to give birth at the reference hospital; Spanish Caucasian origin; 18–45 years of age; singleton pregnancy; non-assisted conception; and normal echography at 20 weeks of gestation (no major malformations). The exclusion criteria included an existing chronic disease; pregnancy complications (except gestational diabetes and hypertensive disorders); and not intending to deliver in the reference hospital. Among the 1350 women invited to participate, 738 (54%) were finally enrolled in the study, of which 665 completed the dietary information

The study protocol was reviewed and approved by the Ethics Committee of the Virgen de la Arrixaca University Clinical Hospital in accordance with the guidelines of The Declaration of Helsinki. Written informed consent was obtained from parents at recruitment. 

### 2.2. Maternal Dietary Intake Assessment and Development of Mediterranean Diet Scores

The dietary information on usual daily food intake was collected at 20 weeks of gestation, using a Food Frequency Questionnaire (FFQ) previously validated among pregnant women of the INfancia y Medio Ambiente—(environment and childhood) (INMA) prospective cohort study [26] which was administered by trained interviewers. The FFQ included 123 items, of which 112 were semi-quantitative, to assess usual food and nutrient intakes during the first 20 weeks of gestation, and 11 qualitative to collect information about the use of dietary supplements and organic food consumption. For each food item, the questionnaire asked how often, on average, the participants had consumed a particular amount of specific type of food from the beginning of pregnancy until the time of the interview. For each food item, standard units or reference serving sizes were specified. The questionnaire had nine possible intake frequency categories, ranging from “less than once per month or never” to “6 or more times per day”. Nutrient values and energy intakes were obtained from the US Department of Agriculture Food Composition Tables [27], as well as other published sources for Spanish foods, portion sizes, and their content for some specific nutrients such as folic acid [28,29]. The intake frequency for each food item was converted to the average daily intake for each participant. For the calculation of the different scores in each of the dietary patterns described below, the consumption of vitamin or mineral supplements by the mother during pregnancy was not considered.

### 2.3. Diet Quality Scores

To evaluate the degree of adherence to the MD during pregnancy, we used two scores: the Alternative Mediterranean Diet score (aMED [30]) and the Relative Mediterranean Diet score (rMED [31]); both are a modified version of the Mediterranean Diet Score (MDS) proposed by Trichopoulou and colleagues in 1995 [32]. As Chatzi et al. reported in different publications [7,33], we did not include alcohol consumption to calculate the scores because the population in the present study involved pregnant women and both scores had been developed for adults. Table 1 shows the main items of each of the scores used to determine adherence to MD, as well as their main differences.

The relative Mediterranean Diet score (rMED) was proposed by Bukcland et al. (2009) for the European Prospective Investigation into Cancer and Nutrition (EPIC)-Spain cohort [31]. This indicator has eight components (originally nine, but alcohol was excluded): vegetables (excluding potatoes), fruits, nuts and seeds (but excluding fruit juices), cereals (including whole grain, refined flour, pasta, rice, and bread), legumes, fish and seafood, dairy products (including low and high fat products, cheese, yogurt, and cream desserts), total meat (including white, red, and processed meat), and olive oil. In addition, before assigning the scores to the rMED groups (except alcohol), the intakes were transformed into grams per 1000 Kcals/day. To assign the scores, tertiles were used instead of the medium, and the values 0, 1, and 2 were assigned to the first, second, and third tertiles of intake, except for meats and dairy products that were assigned to the contrary, the highest tertile being scored with a 0. The possible scores ranged from 0 units (minimal adherence) to 16 units (maximum adherence). An rMED score of 0–5 was labelled as “low”, 6–11 as “medium”, and 12–16 as “high” MD adherence by calculating the corresponding tertiles.

The aMED is also a version adapted by Fung and colleagues in 2005 [30] and used by Chazti et al. (2013) [7] in different publications. This indicator has eight components (nine originally) and a range from 0 to 8. For the beneficial components (vegetables, fruits, fish and seafood, nuts, legumes, and whole cereals), women whose consumption was below the median (cohort-specific median) were assigned a value of 0, and women whose consumption was at or above the median were assigned a value of 1. For the components presumed to be detrimental (red meat, liver, hamburgers, and processed meats), the computation was inversed. For fat intake (the eight-food category), we used the ratio of daily consumption of mono-unsaturated lipids to saturated lipids. The total MD score was categorized to reflect three levels of adherence: (1) ≤3, low; (2) 4–5, medium; and (3) 6–8, high MD quality in each index separately. The main difference between the two scores was that the aMED separated fruits and nuts into two independent groups and did not take into account the intake of dairy products in the indicator. Additionally, it included only whole grains in the cereal component, and white meat was not included in the group of meats and processed meats. Finally, the ratio of mono-unsaturated to saturated fat was included as a fat source (see Table 1).

The Alternate Healthy Eating Index-2010 (AHEI-2010), which is a variation of the AHEI created by Fung et al. in 2005 [30] and has been associated with lower mortality and lower risk of diseases, was created by Chiuve et al. in 2012 [34] and was also used in our study. It is a measure of diet quality based on American dietary guidelines and with modified recommendations from the US Department of Agriculture [27]. This index originally consists of 11 components, one of them being alcohol consumption, and a range from 0 to 110. When it comes to applying this index to our study (pregnant women), an adaptation of the AHEI-2010 was performed eliminating the alcohol component. Due to this modification, the score range changed from 0 to 100 points, as each of the components, 10 in total (instead of 11), contribute with 10 possible points (See Table S1). For intermediate intakes, the proportional part between 0 and 10 was calculated by multiplying the number of daily rations consumed by 10 and then dividing by the criterion for a maximum score for that food group [35]. For example, for zero servings of fruit per day, a score of 0 was assigned; for one serving per day, a score of 2.5 was assigned as four servings per day was considered to be ideal. In this index, as in the case of the aMED, only the whole grain was considered within the group of cereals; the nuts, instead of being included in the fruit component, were included together with the legumes. Regarding meats, the component was composed of red meat and processed meats. Finally, polyunsaturated fats were divided into several items, and sodium consumption was considered. For the transformation of the continuous variable into three degrees of adherence, the quintiles of the scores of the study population were calculated and the three intervals were established: low (42–58 points), medium (59–64 points), and high (65–81 points).

### 2.4. Potential Determinants of Adherence to Mediterranean Dietary Patterns

Information on the following sociodemographic characteristics and lifestyle patterns, which have an established or a potential association with a lower level of adherence to a MD in pregnancy, was collected through questionnaires administered in person during pregnancy: maternal age; parity (0, nulliparous; vs. 1 or more, no nulliparous); gestational diabetes (yes/no); maternal education level (incomplete secondary or less, complete secondary, and university); maternal social class (defined as maternal occupation during pregnancy by using a widely used Spanish adaptation of the international ISCO88 coding system: I–II, managers/technicians; III, skilled; IV–V, semiskilled/unskilled; and unemployed) [36]; maternal body mass index (BMI kg/m^2^) based on height and pre-pregnancy self-reported weight (kg/m^2^) (categorized as normal BMI < 25, overweight BMI 25–29.99, and obesity BMI ≥ 30), maternal smoking and consumption of alcohol during the first 20 weeks of gestation (yes/no); mother’s residential area (urban, residential, and rural). Additionally, questions about physical activity or sedentary lifestyle during the pregnancy period were included in the questionnaire, focused on sedentary activity time (three possible answers: <1 h a day, 1–2 h per day, or ≥3 h per day), sports activity time (three possible answers: no exercise, up to 1 h per week, or ≥2 h per week), and overall physical activity (self-reported), defined as “sedentary/low active”, “moderately active”, and “strong active”.

### 2.5. Statistical Analysis

Data analysis was performed using RStudio version 1.2.5001 (RStudio Team (2019), Boston, MA) [37]. The median and interquartile range (IQR) were calculated for the quantitative variables of the study, and relative frequency distribution was estimated for the qualitative variables. The non-parametric Kruskal–Wallis test or Mann–Whitney U test was performed for statistical comparisons between the median aMED, rMED, and AHEI-2010 scores of the study population according to the sociodemographic and lifestyle factors of the population. The Spearman correlation test was used to study the correlation between the two MD indices. 

The three indices were modelled as continuous variables through multivariable linear regression as categorical variables and through multivariable logistic regression (low vs. medium-high adherence) analysis to identify factors associated with probability of low MDA (≤3 points aMED; ≤5 points rMED) and low level of adherence to a healthy diet (≤58 points AHEI-2010). A medium and high level of adherence to aMED, rMED, and AHEI-2010 were taken as the reference category. Significance for all the tests carried out was set at *p*- value < 0.05.

## 3. Results

The distribution of baseline characteristics of the mothers participating in the Spanish NELA cohort at 20 weeks of gestation are in Table 2. The median age of the mothers at the time of recruitment was 33 years with a median weight of 62 kg and BMI 23.03 kg/m^2^. A total of 49.6% were primiparous and 8.2% had gestational diabetes. More than a half of the cohort (71.7%) lived in urban areas; 55.6% had completed university studies and 18.8% uncompleted secondary or less education. A total of 16.1% of the mothers reported to smoke during pregnancy, and 5.7% reported alcohol consumption. A total of 37.4% belonged to a high social class, and 20.9% were unemployed. A total of 57.9% of the mothers reported that within their leisure time they spent an average of 1 or 2 h a day doing sedentary activities such as watching television, using a computer, or reading. A total of 56.8% of mothers did not practice any kind of sport, and within the group that did some sport activity, the majority (60.2%) reported a sedentary/low active lifestyle.

**Figure 1:**
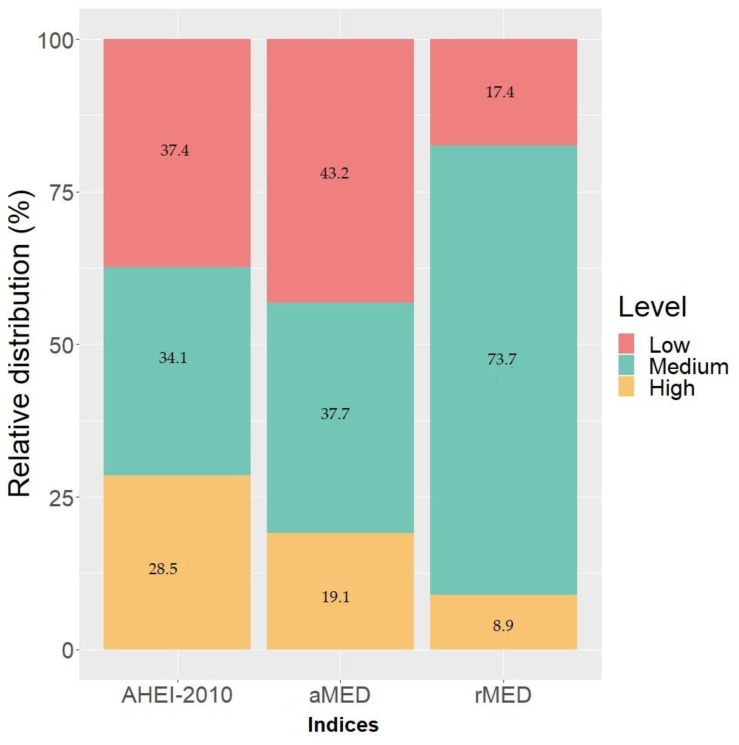
Distribution (%) of the study population according to the degree of adherence to the Mediterranean diet (the Alternative Mediterranean Diet (aMED) and the Relative Mediterranean Diet (rMED)) and degree of adherence to a healthy diet pattern (AHEI-2010) during pregnancy in the Nutrition in Early Life and Asthma (NELA) cohort.

The median adherence of the cohort corresponded to a score of 4.00 (interquartile range (IQR); 3.00–5.00) and 8.00 (IQR; 6.00–10.00) for the aMED and rMED indices, respectively. Both scores on the 3-level categorical scale (low, medium, and high) would be equivalent to a medium level of MDA. Even though there was a significant correlation between the two indices aMED and rMED (Figure S1), significant differences were observed in the MDA distribution when applying the two indices to the study population (Figure 1) (*p* < 0.01). Specifically, in the case of the aMED index, 43.2% and 19.1% of the mothers presented a low and high degree of adherence, respectively, compared to 17.4% and 8.9% when the rMED index was applied.

In our study, the median score obtained by applying the AHEI-2010 index was 61 points (IQR; 55.00–65.00) (Table 2), corresponding to a medium level of adherence. However, 37.4% of the mothers were classified with a low degree of adherence compared to 28.5% of the mothers who were classified with a high adherence to a healthy diet (Figure 1).

Table 3 presents the analysis of the association between sociodemographic and lifestyle factors and the two MDA scores applying (aMED and rMED). The same analysis was carried out applying the AHEI-2010 index (Table 4). When the aMED index was applied, it was observed that the mothers who had an older age, were non-smokers, as well as having a higher educational level and higher social class, and those who practice sports activities ≥2 h per week had a higher level of adherence to MD (*p* < 0.01). In the case of the rMED index, older non-smoking mothers with a higher educational level and higher social class, who practice sports activities ≥2 h per week, and/or have gestational diabetes had a higher MDA (*p* < 0.05). 

Non-smoking mothers with an older age, a higher educational level and a higher social class, a strong active (self-reported) lifestyle, and/or those practice sports activities ≥2 h per week had a higher AHEI-2010 diet pattern (*p* < 0.05) (Table 4). No association with adherence was observed for the remaining variables, including BMI and sedentary activity.

For aMED and rMED indices, older age and practicing regular physical activity ≥2 h per week were positively associated with MDA scores (see Table 5). The higher the number of previous deliveries, the lower the aMED index score (β: −0.29); (95% IC: −0.49; −0.09; *p* < 0.01). Only in the rMED index, a university educational level of the mother was associated with a better score in rMED index (β: 0.85); (95% IC: 0.22; 1.49; *p* < 0.01). In both indices, a younger age was significantly associated with a higher risk of low MDA. (aMED; OR: 0.92; 95% CI: 0.88–0.96; *p* < 0.01; rMED; OR: 0.88; 95% CI: 0.83–0.93, *p* < 0.01). The number of previous deliveries was directly associated with a greater risk of a low MDA for each previous child (aMED; OR: 1.40; 95% CI: 1.08–1.82; *p* < 0.05; rMED; OR: 1.38; 95% CI: 1.00–1.89, *p* < 0.05). Only for the aMED index was it observed that university education was significantly associated with a lower risk of low MDA (OR: 0.48; 95% CI: 0.28–0.83; *p* < 0.01). Regarding the mother’s sports activity and MDA, in the aMED index a lower risk of low adherence was obtained as the weekly hours of sports activity were ≥2 h per week (OR: 0.54, 95% CI: 0.36–0.81, *p* < 0.01). It was observed that living in residential and rural areas decreased the risk of a low MDA but only in the rMED index.

Regarding the AHEI-2010 (Table 6), a younger age (OR: 0.92; 95% CI: 0.88–0.96, *p* < 0.01) and smoking (OR: 1.70, 95% CI: 1.07–2.70, *p* < 0.05) were associated with a higher risk of a low AHEI-2010 dietary pattern. Regarding sport activity, up to 1 h per week of sports activity (OR: 0.56; 95% CI: 0.32–0.96, *p* < 0.05) decreases the probability of presenting a low AHEI-2010 dietary pattern.

## 4. Discussion

In the current prospective cohort study, three different dietary indices based on the data from an FFQ collected at 20 weeks of pregnancy were calculated. In relation to the sociodemographic and lifestyle factors, we found a markedly protective effect on age for the three dietary indices. The older they are, the higher the chance of a good MDA or a diet associated with a lower risk of cardiovascular disease. Additionally, the educational level of the mother and fewer previous deliveries were other variables with a protective effect for MDA, decreasing the probability of poor adherence. These protective markers coincide with those observed by other authors [23,38]. In other studies, the authors observed that a healthy dietary pattern in pregnant women was positively associated with older age, a higher educational level, a higher social class, and greater physical activity [23,39,40,41,42]. Other authors also observed a negative association between the number of previous deliveries and a healthy diet pattern [39,41,42]. In relation to smoking and BMI, contradictory results have been reported. In certain studies, smoke and BMI have been negatively associated with a healthy diet pattern [41,42], while in others overweight/obesity was positively associated [39].

Despite the high degree of correlation that has been observed between the two MD indices (aMED and rMED), statistically significant differences have been observed in the distributions of three groups into which the population can be divided according to their degree of adherence. In general, while previous studies have applied a single index of adherence to study the MD in pregnant women, this is the first study, to our knowledge, in which two different scores have been applied to characterize the MDA in pregnant women along with a third index which represents a healthy diet pattern.

The level of adherence to the MD (aMED) as well as that of the healthy-style diet of pregnant women belonging to our study was in line with that observed in the study carried out by Lange et al. in 2010 [35], whose study population was characterized by presenting a medium level of adherence for both indices (4.6 points in the aMED index and 61 points in the AHEI-2010 index). Our results are also very similar in reference to the percentage of mothers who present a low and high MDA index (using the aMED index) to the results observed by Chatzi et al. in 2012 [43] (42% and 15%, respectively) for a population residing in the Mediterranean area. However, in another study also carried out by Chatzi et al. in 2013 [7], a lower proportion of mothers with a high level of adherence (approx. 7.5% in the population of Valencia) (applying the aMED index) were found, compared to our population (19.1%) despite the proximity of both areas (southern of Spain). On the other hand, we consider it necessary to highlight the trend observed in recent years towards a worsening of the adherence to the MD by the Spanish adult population, who are classified with a low adherence and adopting a less healthy diet (typical of Western countries) [38,44]. It would be interesting to carry out studies to ascertain if this trend is observed in Spanish pregnant women, since it is a period in which mothers tend to be more careful in the choice of foods they eat and avoid certain habits that could be harmful to their health and the health of their offspring.

The differences observed in the distributions of the population according to the MDA between both indices may be due to two issues: (a) the differences in the determination of the foods that make up the single group and (b) the different criteria that exist when trying to differentiate the food groups that conform to the MD pattern, such as olive oil, red meat, dairy products, and cereals. In the case of olive oil, one of the main foods that define MD, the aMED index does not take this ingredient into account as an individual group, while the rMED index does, considering it positive. As a result of these differences, in other cohorts formed in the United Kingdom [45] or the United States [35], where MD is also studied using the aMED index, a lower percentage of low adherence (39.1%) and a higher average MDA value (4.6 points) has been observed, respectively, compared to our cohort, despite being geographic areas where following an MD is not the most common.

It must be considered that our population group has nutritional requirements different from that of a normal adult. Pregnant women suffer certain alterations during the first months of pregnancy that modify the dietary pattern. Moreover, certain foods are also eliminated from the diet, or their intake is reduced because they are not recommended, such as the consumption of fish with high mercury content, while others are encouraged, such as dairy products as a source of calcium. Regarding the last group, dairy products, there is no consensus when it comes to applying the indices or modifying them for pregnant women. Some studies include dairy as a positive food group [7,33,45], while others apply the rMED index without modifications, considering its consumption as negative due to the high fat content and because they do not differentiate between whole and skimmed dairy products, as in our case following the recommendations [6,31,46].

Due to the importance of following a good dietary pattern during pregnancy, it is considered necessary to carry out effective interventions in primary care to promote MDA. Ongoing individual nutritional counseling has been shown to improve nutrition, increasing the intake of dietary fiber, fruits, and vegetables, and reducing the intake of saturated fat [47]. As possible limitations of our study, we must cite the observational nature of the study, with dietary information recorded retrospectively (diet during the first 20 weeks of pregnancy). This may involve some memory bias, possibly reinforced by the social desirability of avoiding the recording of a large intake of unhealthy foods. Such bias would mainly affect mothers with worse eating habits, reducing the variability of the sample and the probability of obtaining significant associations. As possible advantages of our study, the following should be noted: (i) the large sample size and the study population selected from healthy pregnant women in the reference area; thus, the results could be more easily extrapolated to a wider population; (ii) the use of a previously validated FFQ among pregnant women in Spain which may reduce the presence of bias [26]; (iii) the compilation of all the information obtained in the different questionnaires was carried out by trained interviewers; and (iv) the results have been presented for three indices, which allow a better comparability with other studies that use only one index.

## 5. Conclusions

As a general conclusion, in this study pregnant women with a younger age, previous deliveries, low educational level, and who practice unhealthy lifestyles, such as lack of physical activity, are associated with a higher risk of low adherence to the Mediterranean diet (aMED and rMED) and a healthy dietary pattern (AHEI-2010). This fact should be taken into account to design successful educational interventions in the future because, as mentioned above, a healthy diet also has a protective effect on the health of the offspring. The pre-pregnancy and pregnancy periods are an ideal time window to introduce some stimulus that helps to modify dietary and lifestyle habits in a positive way since there is also a very large motivational factor.

## Figures and Tables

**Table 1:** Characteristics of Mediterranean diet adherence pattern indexes applied in pregnancy.

Food Groups	Alternative Mediterranean Score (aMED)	Relative Mediterranean Score (rMED)
Scoring criteria	Ratios/day	Energy density = g*1000 Kcal/day
Vegetables	0 points ≤ median; 1 point > median	Tertile 1 = 0 points; Tertile 2 = 1 point; Tertile 3 = 2 points
Legumes	0 points ≤ median; 1 point > median	Tertile 1 = 0 points; Tertile 2 = 1 point; Tertile 3 = 2 points
Fruit	0 points ≤ median; 1 point > median	(excluding fruit juice) Tertile 1 = 0 points; Tertile 2 = 1 point; Tertile 3 = 2 points
Nuts	0 points ≤ median; 1 point > median	Included in fruit group
Fish	0 points ≤ median; 1 point > median	Tertile 1 = 0 points; Tertile 2 = 1 point; Tertile 3 = 2 points
Cereals	only whole grain 0 points ≤ median; 1 point > median	Tertile 1 = 0 points; Tertile 2 = 1 point; Tertile 3 = 2 points
Meat	(Red and processed meat) 0 points ≥ median; 1 point < median	(All type of meats) Tertile 1 = 2 point; Tertile 2 = 1 point; Tertile 3 = 0 points
Dairy products	Not included	(including skimmed) Tertile 1 = 2 point; Tertile 2 = 1 point; Tertile 3 = 0 points
Mano/Saturated fats ratio	0 points ≥ median; 1 point < median	Not included
Alcohol	Not included	Not included
Potatoes	Included in vegetable groups	Not included
Olive oil cooking	Included in mono/saturated fats ratio group	Tertile 1 = 0 points; Tertile 2 = 1 point; Tertile 3 = 2 points
Poultry	Not included	Included in meat group
Score range	0–9 points	0–16 points
Adherence category	Low = 0–3 points; Medium = 4–5 points; High = ≥ 6 points	Low = 0–5 points; Medium = 6–11 points; High = 12–16 points

**Table 2:** Baseline characteristics of pregnant women at week 20 of pregnancy. NELA birth cohort study (*n* = 665).

	*n*	%	Median	IQR	Min	Max
**Maternal age (years)**			33.00	(30.00–36.00)	18.00	45.00
**Anthropometric measures (*n* = 661)**						
Height (m)			1.64	(1.60–1.68)	1.47	1.82
Weight (kg)			62.00	(56.00–70.00)	40.00	119.00
Body mass index (kg/m^2^)			23.03	(20.83–25.88)	16.23	42.44
Normal weight (< 25)	456	69.0	21.59	(20.25–23.15)	16.23	24.98
Overweight (25–29.99)	143	21.6	26.71	(25.77–28.05)	25.00	29.86
Obese (≥30)	62	9.4	33.89	(31.27–37.16)	30.00	42.44
**Parity, nulliparous**	330	49.6				
**Gestational diabetes (*n* = 649)**	53	8.2				
**Area**						
Urban area	477	71.7				
Residential	96	14.4				
Rural	92	13.8				
**Maternal education**						
Incomplete secondary or less	125	18.8				
Complete secondary	170	25.6				
University	370	55.6				
**Maternal smoking**	107	16.1				
**Maternal alcohol consumption**	38	5.7				
**Maternal social class**						
I–II	249	37.4				
III	146	22.0				
IV–V	131	19.7				
Unemployed	139	20.9				
**Diet index (20 weeks)**						
aMED			4.00	(3.00–5.00)	0.00	8.00
rMED			8.00	(6.00–10.00)	2.00	15.00
AHEI-2010			61.00	(55.00–65.00)	42.00	81.00
**Use of probiotics (*n* = 622)**	108	17.4				
**Sedentary activity time (hours/day) (*n* = 663)**						
<1 h per day	76	11.5				
1–2 h per day	384	57.9				
≥3 h per day	203	30.6				
**Sports activity time (hours/week)**						
Not exercise or sports	378	56.8				
up to 1 h per week	88	13.2				
≥2 h per week	199	29.9				
**Physical activity (self-report)**						
Sedentary/low active	400	60.2				
Moderately active	235	35.3				
Strong active	30	4.5				

IQR: interquartile range.

**Table 3:** Sociodemographic and lifestyle characteristics of women at week 20 of pregnancy according to the Alternative Mediterranean Diet and Relative Mediterranean Diet indexes score distribution. NELA birth cohort study (*n* = 665).

			Alternative Mediterranean Diet (aMED) Index Score (Points)			Relative Mediterranean Diet (rMED) Index Score (Points)		
	*n*	%	*Median*	*IQR*	*p*	*Median*	*IQR*	*p*
**Maternal age (years)**					<0.0001			<0.0001
≥40	46	6.92	4.50	(3.00–5.75)		10.00	(7.25–11.00)	
35–39	191	28.72	4.00	(3.00–5.50)		8.00	(7.00–10.00)	
30–34	288	43.31	4.00	(3.00–5.00)		8.00	(6.00–10.00)	
25–29	109	16.39	3.00	(2.00–5.00)		7.00	(5.00–9.00)	
<25	31	4.66	2.00	(1.00–4.00)		5.00	(4.00–8.00)	
**Body mass index (kg/m^2^) (*n* = 661)**					0.30			0.73
Normal weight (<25)	456	69.0	4.00	(3.00–5.00)		8.00	(6.00–10.00)	
Overweight (25–29.99)	143	21.6	4.00	(2.50–5.00)		8.00	(6.00–10.00)	
Obese (≥30)	62	9.4	3.00	(2.25–5.00)		8.00	(5.25–10.00)	
**Parity (number of previous deliveries)**					0.06			0.66
Nulliparous	330	49.62	4.00	(3.00–5.00)		8.00	(6.00–10.00)	
One or more previous deliveries	335	50.38	4.00	(3.00–5.00)		8.00	(6.00–10.00)	
**Gestational diabetes (*n* = 649)**					0.25			0.028
No	596	91.83	4.00	(3.00–5.00)		8.00	(6.00–10.00)	
Yes	53	8.17	4.00	(3.00–6.00)		9.00	(7.00–11.00)	
**Area**					0.21			0.50
Urban area	477	71.73	4.00	(3.00–5.00)		8.00	(6.00–10.00)	
Residential area	96	14.44	4.00	(3.00–5.00)		8.00	(7.00–10.00)	
Rural	92	13.83	4.00	(2.75–5.00)		8.00	(6.75–10.00)	
**Maternal education**					<0.0001			<0.0001
Incomplete secondary or less	125	18.80	3.00	(2.00–4.00)		7.00	(5.00–9.00)	
Complete secondary and superior	170	25.56	4.00	(2.25–5.00)		7.50	(6.00–9.00)	
University	370	55.64	4.00	(3.00–5.00)		8.50	(7.00–10.00)	
**Maternal social class**					<0.0001			<0.0001
I–II	239	35.94	4.00	(3.00–6.00)		8.00	(7.00–10.00)	
III	150	22.56	4.00	(3.00–5.00)		8.00	(6.00–10.00)	
IV–V	127	19.10	3.00	(2.00–5.00)		8.00	(6.00–9.00)	
Unemployed	149	22.41	3.00	(2.00–5.00)		7.00	(5.00–9.00)	
**Maternal Smoking**					0.009			0.001
No	558	83.91	4.00	(3.00–5.00)		8.00	(6.00–10.00)	
Yes	107	16.09	3.00	(2.00–5.00)		7.00	(5.00–9.00)	
**Maternal alcohol consumption**					0.20			0.17
No	627	94.29	4.00	(3.00–5.00)		8.00	(6.00–10.00)	
Yes	38	5.71	3.50	(2.00–4.75)		7.00	(6.00–9.00)	
								
**Use of probiotics (*n* = 622)**					0.16			0.10
No	514	82.64	4.00	(3.00–5.00)		8.00	(6.00–10.00)	
Yes	108	17.36	4.00	(3.00–5.00)		8.00	(7.00–10.25)	
**Sedentary activity time (hours) (*n* = 663)**					0.31			0.16
<1 h per day	76	11.5	4.00	(3.00–5.00)		8.00	(6.00–10.00)	
1–2 h per day	384	57.9	4.00	(3.00–5.00)		8.00	(6.75–10.00)	
≥3 h per day	203	30.6	4.00	(2.00–5.00)		8.00	(6.00–10.00)	
**Sports activity time (hours)**					<0.0001			<0.0001
No exercise or sports	378	57.1	3.00	(2.00–5.00)		8.00	(6.00–9.00)	
Up to 1 h per week	88	13.3	4.00	(2.00–5.00)		8.00	(6.00–10.00)	
≥2 h per week	199	30.1	5.00	(3.00–6.00)		9.00	(7.00–11.00)	
**Physical activity (self-report)**					<0.0001			0.05
Sedentary/low active	400	60.4	4.00	(2.00–5.00)		8.00	(6.00–10.00)	
Moderately active	235	35.5	4.00	(3.00–5.00)		8.00	(6.00–10.00)	
Strong active	30	4.5	5.00	(4.00–6.00)		8.50	(6.00–10.75)	

**Table 4:** Sociodemographic and lifestyle characteristics of women at week 20 of pregnancy according to the Alternative Healthy Eating Index 2010 score distribution. NELA birth cohort study (*n* = 665).

	Alternative Healthy Eating Index Score (AHEI-2010)		
	Median	IQR	*p*
**Maternal age (years)**			<0.0001
≥40	63.00	(59.00–66.75)	
35–39	62.00	(57.00–66.00)	
30–34	61.00	(56.00–66.00)	
25–29	58.00	(55.00–63.00)	
<25	55.00	(52.50–62.50)	
**Body mass index (kg/m^2^) (*n* = 661)**			1.00
Normal weight (<25)	61.00	(55.00–66.00)	
Overweight (25–29.99)	61.00	(56.00–65.00)	
Obese (≥30)	60.00	(55.00–65.00)	
**Parity (number of previous deliveries)**			0.05
Nulliparous	60.00	(55.00–65.00)	
One or more previous deliveries	61.00	(56.00–66.00)	
**Gestational diabetes (*n* = 649)**			0.66
No	61.00	(56.00–66.00)	
Yes	60.00	(55.00–64.00)	
**Area**			0.23
Urban area	60.00	(55.00–65.00)	
Residential area	62.00	(57.75–65.00)	
Rural	62.00	(56.00–66.00)	
**Maternal education**			<0.0001
Incomplete secondary or less	58.00	(54.00–64.00)	
Complete secondary and superior	59.50	(55.00–64.00)	
University	62.00	(57.00–66.00)	
**Maternal social class**			0.002
I–II	62.00	(58.00–66.00)	
III	60.50	(55.25–65.00)	
IV–V	59.00	(54.00–64.00)	
Unemployed	60.00	(55.00–65.00)	
**Maternal Smoking**			<0.0001
No	61.00	(56.00–66.00)	
Yes	58.00	(54.00–63.00)	
**Maternal alcohol consumption**			0.08
No	61.00	(56.00–65.50)	
Yes	59.50	(53.25–63.75)	
**Use of probiotics (*n* = 622)**			0.51
No	61.00	(56.00–66.00)	
Yes	60.00	(55.00–64.25)	
**Sedentary activity time (hours) (*n* = 663)**			0.65
<1 h per day	62.00	(55.75–66.00)	
1–2 h per day	61.00	(56.00–65.00)	
≥3 h per day	61.00	(55.00–65.00)	
**Sports activity time (hours)**			0.014
No exercise or sports	60.00	(55.00–65.00)	
Up to 1 h per week	61.00	(58.00–66.00)	
≥2 h per week	62.00	(56.00–66.00)	
**Physical activity (self-report)**			0.019
Sedentary/low active	60.50	(56.00–65.00)	
Moderately active	61.00	(55.00–66.00)	
Strong active	64.00	(61.25–67.00)	

Bold values mean “statistical significance” (*p*-value < 0.05). Numbers were expressed as median and interquartile range (IQR) for the quantitative variables. Statistical caparisons using non-parametric Mann–Whitney U test or Kruskal–Wallis test.

**Table 5:** Association between sociodemographic characteristics and life style factors and low adherence to the Mediterranean diet in pregnant women at 20 weeks of gestation (*n* = 659) in the NELA cohort study.

	aMED						rMED					
	β ^†^	(95% CI)		OR ^‡^	(95% CI)		β ^†^	(95% CI)		OR ^‡^	(95% CI)	
**Maternal age (years) ^§^**	0.09	(0.06; 0.12)	**	0.92	(0.88–0.96)	**	0.14	(0.09; 0.19)	**	0.88	(0.83–0.93)	**
**Body mass index (kg/m^2^)**												
Normal weight (<24.99)	Ref			Ref			Ref			Ref		
Overweight (25–29.9)	−0.08	(−0.41; 0.25)		1.07	(0.70–1.61)		0.08	(−0.39; 0.55)		0.83	(0.47–1.44)	
Obese (≥30)	0.10	(−0.36; 0.56)		1.07	(0.60–1.93)		0.15	(−0.52; 0.82)		1.28	(0.64–2.50)	
**Parity (number of previous deliveries) ^§^**	−0.29	(−0.49; –0.09)	**	1.40	(1.08–1.82)	*	−0.20	(−0.48; 0.09)		1.38	(1.00–1.89)	*
**Area**												
Urban area	Ref			Ref			Ref			Ref		
Residential area	−0.08	(−0.47; 0.30)		1.05	(0.64–1.72)		−0.08	(−0.63; 0.47)		0.41	(0.17–0.88)	*
Rural	−0.16	(−0.54; 0.22)		1.08	(0.67–1.76)		0.41	(−0.14; 0.96)		0.41	(0.19–0.80)	*
**Maternal education**												
Incomplete secondary or less	Ref			Ref			Ref			Ref		
Complete secondary and superior	0.21	(−0.21; 0.62)		0.67	(0.40–1.13)		0.10	(−0.50; 0.70)		0.93	(0.50–1.73)	
University	0.37	(−0.08; 0.81)		0.48	(0.28–0.83)	**	0.85	(0.22; 1.49)	**	0.80	(0.40–1.59)	
**Maternal social class**												
I–II	Ref			Ref			Ref			Ref		
III	−0.14	(−0.53; 0.24)		1.01	(0.61–1.64)		0.06	(−0.49; 0.62)		1.26	(0.62–2.52)	
IV–V	−0.24	(−0.69; 0.20)		1.09	(0.62–1.92)		0.02	(−0.63; 0.66)		1.19	(0.55–2.51)	
Unemployed	−0.08	(−0.51; 0.35)		1.08	(0.63–1.86)		−0.12	(−0.74; 0.50)		1.30	(0.63–2.68)	
**Maternal Smoking, yes**	−0.02	(−0.39; 0.36)		0.94	(0.59–1.51)		−0.17	(−0.71; 0.36)		1.11	(0.63–1.92)	
**Maternal alcohol consumption, yes**	−0.23	(−0.80; 0.33)		1.19	(0.58–2.44)		−0.31	(−1.13; 0.50)		1.18	(0.46–2.75)	
**Sedentary activity time (hours)**												
<1 hour per day	Ref						Ref			Ref		
1–2 hours per day	−0.17	(−0.60; 0.26)		1.30	(0.76–2.28)		0.05	(−0.57; 0.67)		0.73	(0.36–1.53)	
≥ 3 hours per day	−0.28	(−0.75; 0.19)		1.35	(0.74–2.47)		−0.19	(−0.86; 0.49)		1.42	(0.68–3.11)	
**Sports activity time (hours)**												
Not exercise or sports	Ref			Ref			Ref			Ref		
up to 1 hour per week	0.08	(−0.32; 0.49)		0.69	(0.41–1.14)		−0.01	(−0.59; 0.57)		1.36	(0.72–2.49)	
≥ 2 hour per week	0.56	(0.25; 0.87)	**	0.54	(0.36–0.81)	**	0.82	(0.37; 1.27)	**	0.77	(0.43–1.35)	
**Physical activity (self-report)**												
Sedentary/ low active	Ref			Ref			Ref			Ref		
Moderately active	0.23	(−0.05; 0.52)		0.71	(0.49–1.01)		0.09	(−0.32; 0.50)		0.89	(0.54–1.44)	
Strong active	0.90	(0.25; 1.56)	**	0.45	(0.17–1.10)		0.10	(−0.85; 1.05)		0.59	(0.13–1.95)	

Ref: Reference; CI: confidence interval; β: Regression coefficients; OR: Odds ratio. ^§^ Introduced as a continuous variable. aMED (Alternative Mediterranean Diet index score). rMED (Relative Mediterranean Diet index score). ^†^ Multivariate linear regression analysis. Indices were modelled as continuous variables. ^‡^ Multivariate logistic regression analysis. Indices were modelled as categorical variables (medium-high adherence vs low); aMED score: low (≤3 points) and medium-high (≥4 points); rMED score: low (≤5 points) and medium-high (≥6 points). All models were adjusted for age, BMI, parity, residential area, maternal education and social class, smoking and alcohol consumption, sedentary activity time, sport activity time and physical activity. * *p*-value < 0.05; ** *p*-value < 0.01.

**Table 6:** Association between sociodemographic characteristics and lifestyle factors and low adherence to a healthy diet pattern. Alternative Healthy Eating Index (AHEI-2010) in pregnant women at 20 weeks gestation (*n* = 659) in the NELA cohort study.

	β ^†^	(95% CI)		OR ^‡^	(95% CI)	
**Maternal age (years) ^§^**	0.31	(0.18; 0.44)	**	0.92	(0.88–0.96)	**
**Body mass index (kg/m^2^)**						
Normal weight (<24.99)	Ref			Ref		
Overweight (25–29.9)	0.43	(−0.88; 1.74)		0.90	(0.59–1.37)	
Obese (≥30)	0.82	(−1.04; 2.67)		0.82	(0.44–1.50)	
**Parity (number of previous deliveries) ^§^**	−0.24	(−1.04; 0.55)		1.01	(0.78–1.30)	
**Area**						
Urban area	Ref			Ref		
Residential area	0.77	(−0.76; 2.30)		0.78	(0.46–1.29)	
Rural	0.83	(−0.70; 2.35)		0.67	(0.40–1.10)	
**Maternal education**						
Incomplete secondary or less	Ref			Ref		
Complete secondary and superior	1.10	(−1.76; 1.57)		0.83	(0.49–1.40)	
University	0.75	(−1.02; 2.51)		0.70	(0.40–1.23)	
**Maternal social class**						
I–II	Ref			Ref		
III	−0.49	(−2.03; 1.05)		1.54	(0.93–2.54)	
IV–V	−1.23	(−3.02; 0.55)		1.68	(0.95–3.00)	
Unemployed	−0.17	(−1.89; 1.56)		1.11	(0.62–1.95)	
**Maternal Smoking, yes**	−1.26	(−2.74; 0.23)		1.70	(1.07–2.70)	*
**Maternal alcohol consumption, yes**	−1.49	(−3.75; 0.77)		1.34	(0.65–2.76)	
**Sedentary activity time (hours)**						
<1 h per day	Ref			Ref		
1–2 h per day	0.10	(−1.61; 1.82)		0.82	(0.43–1.56)	
≥3 h per day	0.15	(−1.74; 2.03)		1.16	(0.63–2.16)	
**Sports activity time (hours)**						
Not exercise or sports	Ref			Ref		
up to 1 h per week	1.36	(−0.26; 2.97)		0.56	(0.32–0.96)	*
≥2 h per week	0.80	(−0.45; 2.05)		0.72	(0.48–1.08)	
**Physical activity (self-report)**						
Sedentary/low active	Ref			Ref		
Moderately active	−0.19	(−1.33; 0.95)		1.54	(1.07–2.23)	*
Strong active	2.64	(0.02; 5.27)	*	0.35	(0.10–1.00)	

Ref: Reference; CI: confidence interval; β: Regression coefficients; OR: Odds ratio. AHEI-2010 (Alternative Healthy Index score). ^§^ Introduced as a continuous variable. _†_ Multivariate linear regression analysis. Indices were modelled as continuous variable. ^‡^ Multivariate logistic regression analysis. Indices modelled as categorical variable (medium-high vs low adherence); AHEI-2010 score: low (≤58 points) and medium-high (>58 points). Both models were adjusted for age, BMI, parity, residential area, maternal education and social class, smoking and alcohol consumption, sedentary activity time, sport activity time and physical activity. * *p*-value < 0.05; ** *p*-value < 0.01.

## Data Availability

The authors declare that they have followed the protocols of their work center on the publication of patient data and that all the patients included in the study have received sufficient information and have given their informed consent in writing to participate in that study.

## Supplementary Materials

The following are available online at https://www.mdpi.com/2072-6643/13/4/1248/s1, Figure S1: Correlation study between the three indices used to evaluate the pattern of adherence to the Mediterranean diet (aMED and rMED) and adherence to a healthy diet pattern (AHEI-2010) in the NELA cohort. Table S1: The AHEI-2010 scoring method and mean scores at baseline among pregnant women in the NELA cohort Study.

## NELA Study Group

ME Candel-Torralba ^1^, L Garcia-Marcos (PI) ^1,2,4,18^, MJ Gimenez-Banon ^1^, A Martinez-Torres ^1,2,18^, E Morales (PI) ^1,3,^ V Perez-Fernandez ^1,4,18^, M Sanchez-Solis ^1,2,4,18^, A Nieto ^1,5^, MT Prieto-Sanchez ^1,5^, M Sanchez-Ferrer ^1,5^, L Fernanez-Palacios ^1,6^, VP Gomez-Gomez ^1,6^, C Martinez-Graciá ^1,6^, P Peso-Echarri ^1,6^, G Ros-Berruezo ^1,6^, M Santaella-Pacua ^l1,6^, A Gazquez ^1,7^, E Larque ^1,7^, MT Pastor-Fajardo ^1,7^, M Sanchez-Campillo ^1,7^, A Serrano-Munuera ^1,7^, M Zornoza-Moreno ^1,7^, P Jimenez-Guerrero ^1,8^, E Adomnei ^1,9^, JJ Arense-Gonzalo ^1,9^, J Mendiola ^1,9^, F Navarro-Lafuente ^1,9^, AM Torres-Cantero ^1,9^, C Salvador-Garcia ^10^, M Segovia-Hernández ^1,11^, G Yagüe-Guirao ^1,11^, PL Valero-Guillén ^1,12^, FV Aviles-Plaza ^1,13^, J Cabezas-Herrera ^1,13^, A Martinez-Lopez ^1,13^, M Martinez-Villanueva ^1,13^, JA Noguera-Velasco ^1,13^, A Franco-Garcia ^1,14^, AM Garcia-Serna ^1,14^, T Hernandez-Caselles ^1,14,18^, E Martin-Orozco ^1,14,18^, M Norte-Muñoz ^1,14^, M Canovas ^1,14^, E Cantero-Cano ^1^, T de Diego ^1,14^, JM Pastor ^1,14^, RA Sola-Martínez ^1,14^, A Esteban-Gil ^1,17^, JT Fernández-Breis ^1,15^, MV Alcántara ^16^, S Hernández ^16^, C López-Soler ^16^.

^1^ Biomedical Research Institute of Murcia, IMIB-Arrixaca, Murcia, Spain; ^2^ Paediatric Respiratory Unit, “Virgen de la Arrixaca” Children’s University Clinical Hospital, University of Murcia, Spain; ^3^ Department of Public Health Sciences, University of Murcia, Spain; ^4^ Department of Paediatrics, University of Murcia, Spain; ^5^ Obstetrics & Gynaecology Service, “Virgen de la Arrixaca” University Clinical Hospital, University of Murcia, Spain; ^6^ Food Science and Technology Department, Veterinary Faculty of Veterinary, University of Murcia, Spain; ^7^ Department of Physiology, Faculty of Biology, Campus Mare Nostrum. University of Murcia, Spain; ^8^ Regional Atmospheric Modelling Group, Department of Physics, University of Murcia, Spain; ^9^ Department of Public Health Sciences, University of Murcia, Spain; ^10^ Microbiology Service, General University Hospital Consortium, University of Valencia, Spain; ^11^ Microbiology Service, University Clinical Hospital “Virgen de la Arrixaca”, University of Murcia, Spain; ^12^ Microbiology and Genetics Department, University of Murcia, Spain; ^13^ Molecular Therapy and Biomarkers Research Group, Clinical Analysis Service, University Clinical Hospital “Virgen de la Arrixaca”, University of Murcia, Spain; ^14^ Department of Biochemistry and Molecular Biology B and Immunology, University of Murcia, Spain; ^15^ Department of Informatics and Systems, University of Murcia, Spain; ^16^ Paediatric and Adolescent Clinical Psychology University Research Group (GUIIA-PC), University of Murcia, Spain; ^17^ Foundation for Healthcare Training & Research of the Region of Murcia (FFIS); ^18^ Network of Asthma and Adverse and Allergic Reactions (ARADyAL).
