# Supplementary material for: Adherence to the Mediterranean Diet and Determinants Among Pregnant Women: The NELA Cohort
Source: Nutrients. 2021 Apr 10;13(4):1248. doi: 10.3390/nu13041248 (PMC8070446; doi:10.3390/nu13041248)
Supplement: Supplementary file 1 [file nutrients-13-01248-s001.pdf]

SUPPLEMENTARY DATA

Table S1. The AHEI-2010 scoring method and mean scores at baseline among pregnant women in the NELA cohort Study.

**Component**	**Criteria for minimum score (0)**	**Criteria for maximum score (10)**
Vegetables, *servings/d*	0	≥5
Fruit, *servings/d*	0	≥4
Whole grains, *g/d*	0	75
Sugar-sweetened beverages and fruit juice, *servings/d*	≥1	0
Nuts and Legumes, *servings/d*	0	≥1
Red and processed meat, servings/d	≥1.5	0
Trans Fat, % of energy	≥4	≤0.5
Long-chain (n-3) fats (EPA* + DHA**), *mg/d*	0	250
PUFA‡, *% of energy*	≤2	≥10
Sodium, *mg/d*	Highest decile	Lowest decile

EPA: Eicosapentanoic acid. DHA: Docosahexaenoic acid. PUFA: Polyunsaturated fatty acid.

SUPPLEMENTARY DATA

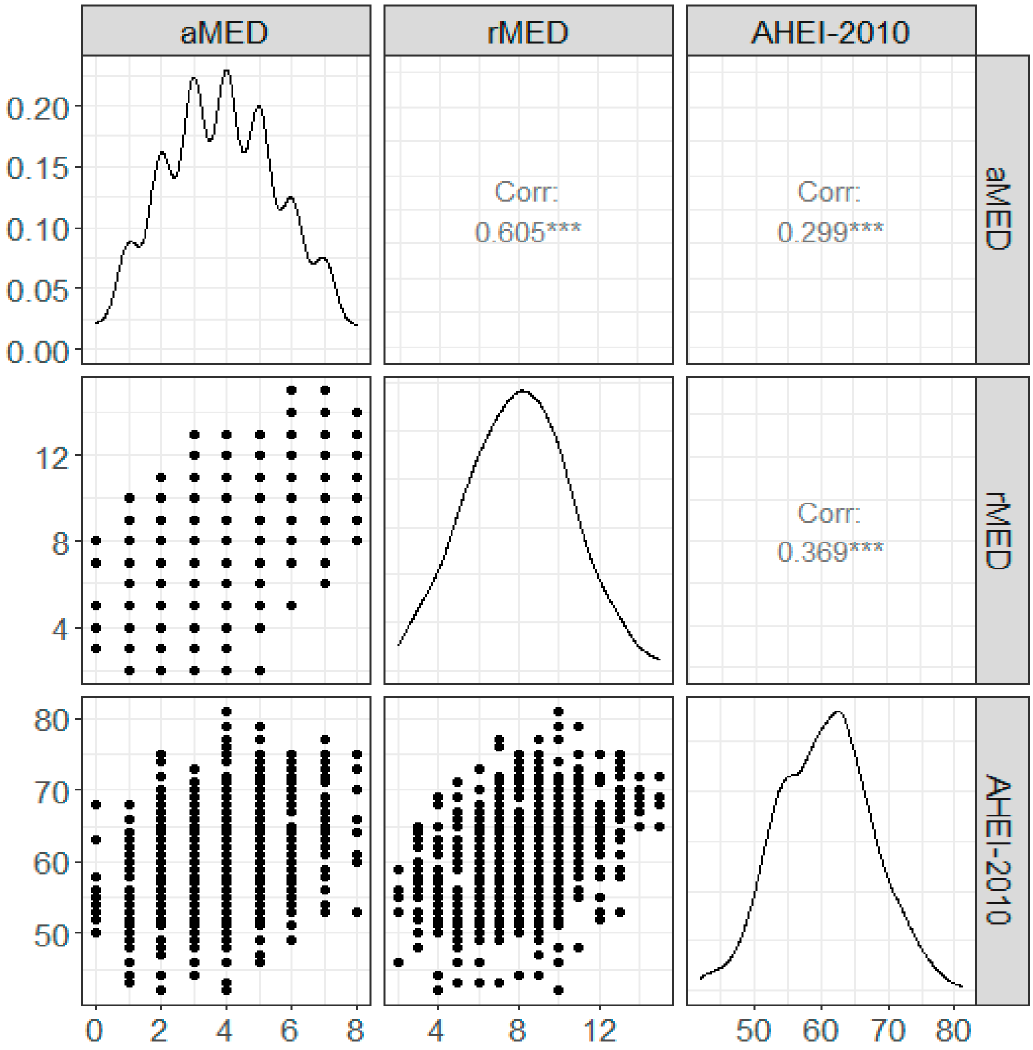

Figure S1. Correlation study between the 3 indices used to evaluate the pattern of adherence to the Mediterranean diet (aMED and rMED) and adherence to a healthy diet pattern (AHEI-2010) in the NELA cohort. *** (p-value<0.001); Corr: degree of correlation among indices.
